# New York and New England Association of Railway Surgeons

**Published:** 1911-11

**Authors:** F. A. Goodwin, George Chaffee

**Affiliations:** President; Binghamton, N. Y; 338 47th Street, Brooklyn, N. Y.


### New York and New England Association of Railway Surgeons

THE twenty-first annual session of the New York and New England Association of Railway Surgeons will be held at the Hotel Astor, New York City, on Thursday, November 16th, 1911. A very interesting and attractive program has been arranged. Railway surgeons, attorneys and officials and all members of the medical profession are cordially invited to attend.

DR. F. A. GOODWIN, President, Binghamton, N. Y
DR. GEORGE CHAFFEE, Corresponding Secretary,
338 47th Street, Brooklyn, N. Y.

